# Clinical features associated with the presence of anti-Ro52 and anti-Ro60 antibodies in Jo-1 antibody-positive anti-synthetase syndrome

**DOI:** 10.3389/fimmu.2024.1399451

**Published:** 2024-06-04

**Authors:** Koichi Yamaguchi, Qi Tang, Paul Poland, Daniel P. Reay, Alyssa Gregory, Rohit Aggarwal, Chester V. Oddis, Dana P. Ascherman

**Affiliations:** ^1^ Department of Allergy and Respiratory Medicine, Gunma University Graduate School of Medicine, Maebashi, Japan; ^2^ Division of Rheumatology and Clinical Immunology, Department of Medicine, University of Pittsburgh Medical Center, Pittsburgh, PA, United States; ^3^ Department of Rheumatology and Immunology, Second Xiangya Hospital of Central South University, Changsha, China; ^4^ Department of Surgery, University of Pittsburgh Medical Center, Pittsburgh, PA, United States

**Keywords:** myositis, HRS (histidyl-tRNA synthetase), Ro52, Ro60, antibody

## Abstract

**Introduction:**

Anti-SSA antibodies target two unrelated proteins, Ro52 (E3 ligase) and Ro60 (RNA binding protein). Previous studies indicate that anti-Ro52 antibodies are frequently associated with various myositis-specific autoantibodies (MSAs)–including anti-tRNA synthetase antibodies—and that the coexistence of MSAs and anti-Ro52 antibodies may portend worse clinical outcomes. Although not well-described in the setting of myositis, work from our animal model of HRS (histidyl-tRNA synthetase)-induced myositis suggests that anti-Ro60 antibodies may also be linked to specific MSAs such as anti-HRS/Jo-1. We therefore aimed to demonstrate the prevalence and clinical characteristics of Ro52 and Ro60 antibody positivity in patients possessing Jo-1 antibodies.

**Methods:**

To establish the immunological link between anti-synthetase, anti-Ro52, and anti-Ro60 antibodies, we evaluated the relative titers of these antibodies in blood and bronchoalveolar lavage fluid (BALF) of mice following immunization with HRS/Jo-1. In parallel, we used ELISA-based approaches to assess sera from 177 anti-Jo1 antibody-positive patients for the presence of anti-Ro52 and/or anti-Ro60 antibodies. We then determined statistical associations between co-existing anti-Jo-1, anti-Ro52, and/or anti-Ro60 antibodies and clinical manifestations associated with the anti-synthetase syndrome.

**Results:**

Mice immunized with HRS had higher levels of anti-Ro52 and anti-Ro60 antibodies in serum and BALF than PBS-immunized mice. In 177 anti-Jo-1 antibody-positive patients, the prevalence of anti-Ro52 and anti-Ro60 antibodies was 36% and 15%, respectively. The frequency of dry eye/dry mouth, interstitial pneumonia, and pulmonary events over time differed between patients with various combinations of anti-Ro52 and anti-Ro60 antibodies. While anti-Ro52 antibodies generally correlated with statistically significant increases in each of these clinical manifestations, the presence of Ro60 antibodies alone was associated with decreased frequency of ILD.

**Discussion:**

Anti-Ro52 and/or anti-Ro60 antibodies are often co-expressed with anti-Jo1 antibodies, defining clinical subsets with different disease course/outcomes.

## Introduction

Idiopathic inflammatory myopathies (IIMs) are rare systemic autoimmune diseases characterized by progressive muscle weakness and distinct skin rashes as well as high frequency of lung involvement. Often marked by specific autoantibodies, IIMs encompass various clinical subtypes including polymyositis (PM), dermatomyositis (DM), and necrotizing myopathy (1). Autoantibodies in myositis consist of myositis-associated antibodies (MAAs) and myositis-specific antibodies, of which anti-tRNA synthetase (ARS) antibodies represent a prominent subset (2). Of the 8 known anti-ARS antibodies, anti-Jo1 antibody is the most common, accounting for 15–30% of patients with myositis (3, 4). Patients with anti-Jo-1 antibodies typically present with some combination of muscle weakness, arthritis, Raynaud’s, mechanic’s hands, and/or interstitial lung disease (ILD)—but have a better prognosis than those with non-anti-Jo-1 ARS antibodies (5, 6). Notably, anti-Ro/SSA antibody, one of the MAAs, often coexists with anti-ARS antibodies and other MAAs (2). The SSA antigens include Ro52 and Ro60 proteins that are not structurally or functionally related; while Ro52 is a member of the tripartite motif family of proteins (E3 ligase), Ro60 ensures quality-control for misfolded RNA (7). In turn, these autoantigens are associated with different autoimmune processes: anti-Ro60 antibodies are mainly detected in systemic lupus erythematosus (SLE) and Sjögren syndrome (SS), while anti-Ro52 antibodies are present in a number of autoimmune diseases ranging from SLE and SS to IIM (8).

In terms of phenotypic associations, it is well-recognized that patients with both anti-ARS and anti-Ro52 antibodies may develop more aggressive ILD and even pulmonary fibrosis (9–11). Beyond the severity of ILD, the coexistence of anti-Ro52 and anti-Jo-1 antibodies has been linked to other clinical findings such as mechanic’s hands and malignancy (12, 13). However, much less is known about the prevalence and clinical associations of anti-Ro60 antibodies in the context of the anti-synthetase syndrome. Based on observations in our well-established mouse model of HRS (histidyl-tRNA synthetase)-induced myositis (14–16) in which mice immunized with recombinant HRS (Jo-1) protein develop anti-Ro60 as well as anti-Ro52 antibodies, we sought to define the prevalence of anti-Ro60 antibodies (with/without co-existing anti-Ro52 antibodies) and corresponding clinical features in humans with anti-Jo-1 antibody-positive anti-synthetase syndrome. At the same time, we used our murine model of the anti-synthetase syndrome to clarify potential mechanisms underlying the co-development of anti-Jo-1 and anti-Ro52/60 antibodies in human disease.

## Methods

### Mice

The immunization protocols utilized in this study involved female C57BL/6 mice (Jackson Laboratory; Bar Harbor, Maine) between the ages of 8 and 10 weeks, matching our previously published studies (14–16) and paralleling the female predominance of IIM. The Institutional Animal Care and Use Committee of the University of Pittsburgh approved these immunization protocols that were based on intramuscular (IM) administration of recombinant histidyl-tRNA synthetase (HRS) protein, as previously described (16).

### Recombinant antigen

In accordance with prior description (17), we generated the amino-terminal fragment of murine HRS as a maltose binding protein [MBP] fusion protein designated MA/MBP, which consists of amino acids 1–151 of murine HRS fused to the carboxy-terminal end of MBP (heretofore referenced as recombinant HRS). Where indicated, we used an MBP control protein produced from the same vector system (pMALc2; New England Biolabs, Ipswich, Massachusetts) without any additional sequence insertion. To mitigate potential experimental variability associated with fluctuations in protein quality, we purified multiple batches of expressed proteins using amylose resin according to the manufacturer’s protocol (New England BioLabs, Ipswich, Massachusetts), followed by dialysis in phosphate-buffered saline (PBS) and filter sterilization. Recombinant Ro52 (ProSpec-Tany TechnoGene Ltd, Ness‐Ziona, Israel) and Ro60 (US Biological, Salem, Massachusetts) were obtained from commercial sources, as indicated.

### Mouse immunization

In our study, experimental mice received IM injections of IFA emulsions [administered to both hamstrings in a total volume of 150 μl (75 μl per side)]] containing the TLR7/8 agonist R848 (Invivogen, San Diego, California; 0.1 μg/ml) and either MA/MBP (1.2 mg/ml) or phosphate-buffered saline (PBS). Two weeks post-immunization, mice in both experimental groups received additional intramuscular R848 solubilized in 100 μl PBS (0.2 μg/ml). After an additional 6 weeks, animals were euthanized for collection of bronchoalveolar lavage fluid (BALF), blood/serum, lung tissue, and muscle, as previously described (18–20). The timeline for these studies is outlined in **Supplementary Figure 1**.

### *In vivo* administration of cigarette smoke

A five-chamber smoking apparatus designed for targeted smoke delivery in restrained mice was maintained in a monitored, flow-regulated fume hood (21). Designated female mice were exposed to cigarette smoke from four unfiltered cigarettes per day (Reference Cigarettes (lot: 1R6F), University of Kentucky, Lexington, KY), 5 days/week for 3 months (starting 4 weeks prior to immunization). Control mice were maintained in the same facility, but did not undergo cigarette smoke exposure.

### Patients

This prospective study examined 177 patients with anti-Jo1 antibody-positive myositis enrolled in the University of Pittsburgh longitudinal myositis registry from 1976 to 2019. PM and DM were diagnosed based on the criteria of Bohan and Peter (22), as many patients were enrolled prior to the development of the revised 2017 ACR/EULAR criteria for IIM (1). Anti-synthetase syndrome was diagnosed in patients with anti-ARS antibodies and specific clinical manifestations (myositis, arthritis, Raynaud’s phenomenon, mechanic’s hands, ILD) according to criteria established in previous studies (3). While patients with features of the anti-synthetase syndrome alone were classified as myositis (23), patients with anti-Jo-1 antibodies who also met specific criteria for SLE (24, 25), scleroderma (26), or rheumatoid arthritis (27) were classified as overlap syndromes (28). Patients meeting criteria for scleroderma (26) or primary Sjogren’s syndrome (29–31) were included as additional controls for this analysis. The University of Pittsburgh Institutional Review Board approved this study (Approval number: 19090054–016) that adhered to tenets of the Declaration of Helsinki.

### Evaluation of clinical findings

Our study examined clinical findings at the time of registry enrollment and corresponding serum collection. Clinical manifestations at baseline evaluation were categorized as follows: muscle weakness (proximal), cutaneous abnormalities (mechanic’s hands, classic DM rashes [that include heliotrope rash, Gottron papule/sign, V-neck sign, and/or shawl sign]), arthritis/arthralgia, vascular dysfunction (Raynaud’s phenomenon), gastrointestinal involvement (upper or lower dysphagia), parenchymal lung abnormalities (interstitial lung disease (ILD)), and sicca symptoms (dry eyes and dry mouth). Patient observation periods spanned the time from study enrollment/collection of serum samples to last visit or death. Pulmonary events included lung transplantation and/or death occurring over a median (IQR) follow up of 6.6 (1.8–10) years (32).

### ELISA-based measurement of anti-Ro52, anti-Ro60, and anti-HRS antibodies

We measured the optical density 450 nm (OD_450_) values of anti-Ro52, anti-Ro60, and anti-murine/human HRS antibodies in mouse sera and BALF as well as in human sera using the following procedures. In mice, we utilized a standard solid-phase enzyme-linked immunosorbent assay protocol to measure the OD_450_ values of anti-Ro52, anti-Ro60, and anti-murine HRS antibodies according to procedures established in our laboratory. Specifically, ninety-six-well microtiter plates (Thermo Fisher Scientific, Roskilde, Denmark) were coated with BSA (2 μg/ml), human Ro52 (2 μg/ml; ProSpec-Tany TechnoGene Ltd, Ness‐Ziona, Israel), human Ro60 (1 μg/ml; US Biological, Salem, Massachusetts) or baculovirus-expressed murine HRS (1μg/ml) in a carbonate buffer (50 mM NaHCO3/Na2CO3, pH 9.6) and incubated overnight at 4°C. Subsequently, the plates were subjected to three washes with PBS containing 0.05% Tween-20. Following these steps, wells were blocked with PBS-0.05% Tween-20 containing 1% BSA for 2 hours at room temperature, and appropriately diluted serum samples (1:500 for Ro52 and Ro60; 1:2500 for murine HRS) or BALF samples (1:16) were then added for a 2-hour incubation period. After sequential washing steps, a subsequent 60-minute incubation with horseradish peroxidase-conjugated goat anti-mouse IgG (1:20,000 for Ro52 and Ro60, 1:10,000 for HRS; Abcam, Massachusetts, USA) was performed. Enzymatic reactions were initiated using 3,3,5,5-Tetramethyl-benzidine (TMB) (Sigma-Aldrich, Missouri, USA) and then stopped with 1 N H2SO4. Color development was measured at 450 nm using a BioTek Synergy H1 microplate reader (Agilent, California, USA) and quantified as adjusted OD_450_ values [OD_450_ with antigen - OD_450_ with BSA]. All assays were conducted in duplicate wells.

For human sera, we followed a procedure similar to that described above, with the following exceptions involving different blocking solutions, antibody dilutions, and substrate antigens. First, the blocking solution used for human serum ELISAs consisted of Tween-20 containing 4% whey protein and 15% goat serum for assessment of anti-Ro52 and anti-Ro60 antibodies versus PBS-0.05% Tween-20 containing 1% BSA for anti-full length human HRS antibody. Second, we used human sera diluted in corresponding blocking buffers to 1:500 for anti-Ro52 and anti-Ro60 antibodies and 1:20,000 for anti-full length human HRS antibodies. Third, the source of recombinant human Ro52 was from Sigma-Aldrich (Missouri, USA). Fourth, in addition to anti-Ro52, anti-Ro60 and anti-full-length HRS antibody responses, we assessed relative titers of antibodies targeting recombinant protein subfragments (expressed as maltose binding protein fusion proteins) comprising the immunodominant amino terminal portion of human HRS (amino acids 1–151) (19). Finally, we used slightly different concentrations of secondary antibody (horseradish peroxidase-conjugated goat anti-human IgG (Abcam, Massachusetts, USA)) diluted in appropriate buffers for anti-Ro52 and anti-Ro60 antibodies (1:5000) versus anti-full length human HRS antibody (1:10,000). Antibody titers were quantified by converting adjusted OD_450_ values (OD_450_ with antigen - OD_450_ without antigen) to standard units using designated reference sera and corresponding dose-response curves (different reference sera were used for anti-Ro52, anti-Ro60, and anti-full-length human HRS antibodies). We established a cutoff for Ro52 and Ro60 antibody positivity based on parallel measurements in healthy controls and then compared serum anti-Ro52 and anti-Ro60 antibody levels between patients with anti-Jo-1 antibodies and selected control groups (healthy controls, primary Sjogren’s syndrome (SS), and patients with non-Jo-1 anti-tRNA synthetase, anti-signal recognition particle (SRP), or anti-topoisomerase I (anti-topo I) antibodies).

### Competition ELISA

In competition ELISAs, human sera were pre-incubated with different concentrations of recombinant HRS-MBP fusion proteins (HA/MBP=human HRS aa 1–151 fused to MBP; Jo-1/MBP=full-length human Jo-1 fused to MBP) for 1 hour at room temperature prior to assessment of anti-Ro52, anti-Ro60, and anti-HRS antibody titers according to the procedures defined above.

### Statistical analysis

Statistical analyses were conducted using SPSS version 27 software (IBM Corp., Armonk, NY, USA). Inter-group comparisons of continuous variables were assessed via the Mann-Whitney U test for non-parametric data. While the Kruskal-Wallis test with Bonferroni correction was used to compare differences in non-parametric data among 3 or more groups, ANOVA was used to evaluate mean values of normally distributed data in multi-group comparisons. Dichotomous outcomes/frequencies were evaluated through Chi-square or Fisher’s exact tests. Spearman rank correlation analysis was used to determine associations between titers of different autoantibodies. Time to pulmonary events was assessed using the Kaplan-Meier method, with statistical significance determined by the log-rank test. Two-tailed p-values <0.05 were considered statistically significant for all analyses.

## Results

### Identification of anti-Ro52 and anti-Ro60 antibodies in mice with HRS-induced myositis

**Figure 1:**
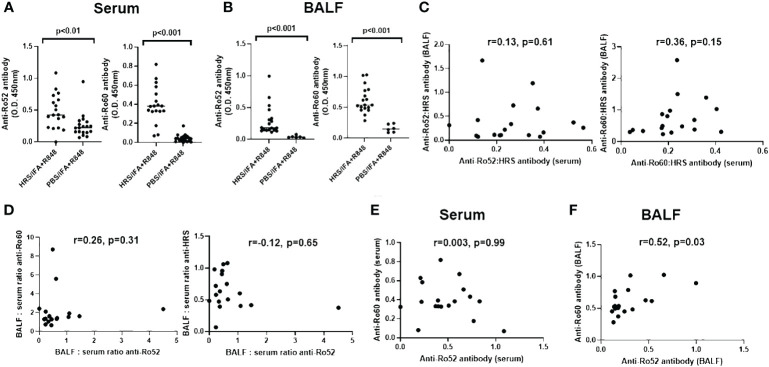
Expression of anti-Ro52 and anti-Ro60 antibodies in blood and bronchoalveolar lavage of HRS-immunized mice. Dot plots in **(A, B)** depict relative titers of anti-Ro52 and anti-Ro60 antibodies (expressed as optical density 450 (OD_450_) values) in serum and bronchoalveolar lavage fluid (BALF) isolated from HRS-immunized mice (n=18) versus PBS-immunized control mice (n=19 serum, n=6 BALF). Bars indicate median OD_450_ values, and p-values based on Mann-Whitney U-testing are listed above each comparison. While **(C)** shows the correlation between anti-Ro52: anti-HRS and anti-Ro60: anti-HRS antibody ratios in BALF versus serum, **(D)** demonstrates the correlation between BALF: serum ratios of anti-Ro52 versus anti-Ro60 and anti-HRS antibody titers (OD_450_ values). **(E, F)** show the correlation between anti-Ro52 and anti-Ro60 antibody titers in serum **(E)** and BALF **(F)** derived from HRS-immunized mice. Correlation coefficients and p-values determined by Spearman’s rank correlation analysis are shown at the top of **(C-F)**. Data depicted for BALF and serum antibody profiles represent pooled experimental groups that received the indicated immunogen, but differed by exposure to cigarette smoke versus air.

To understand the relationship between HRS autoimmunity and the development of antibodies targeting Ro52 and Ro60, we assessed the expression of serum as well as BALF anti-Ro52 and anti-Ro60 antibodies in mice following immunization with recombinant HRS and ongoing exposure to cigarette smoke versus air over a two month period. As shown in **Figure 1A**, mice immunized with HRS and the TLR7/8 agonist R848 demonstrated significantly higher titers of serum anti-Ro52 and anti-Ro60 antibodies compared to the control group immunized with PBS and R848 (p< 0.01 and p<0.001, respectively). In turn, HRS-immunized mice also demonstrated higher levels of anti-Ro52 and anti-Ro60 antibodies in BALF (**Figure 1B**).

Of note, the lack of significant correlation between ratios of anti-Ro52 or anti-Ro60: anti-HRS antibodies in serum and BALF (r values 0.13 and 0.36, respectively (p-values > 0.05); **Figure 1C**) suggested that antibody formation was taking place within the lung as well as the periphery, as the correlation between ratios of BALF and serum antibodies targeting these autoantigens (and the slope of the regression line describing this relationship) would be expected to approach 1 in the case of simple blood-alveolar transit. Paralleling these observations, comparison of BALF: serum anti-Ro52 antibody ratios to BALF: serum ratios of anti-HRS and anti-Ro60 antibodies also suggested differential anti-Ro60 antibody production in the lung and the periphery, with respective correlation coefficients of only -0.12 and 0.26 (p>0.05) (**Figure 1D**). Furthermore, there was no correlation between the serum anti-Ro52 and anti-Ro60 antibody responses (r=0.003, p>0.05; **Figure 1E**), indicating that formation of anti-Ro52 and anti-Ro60 antibodies in *serum* did not represent a simple dose response effect related to intensity of anti-HRS antibody formation. In BALF, on the other hand, there was a modest correlation (r=0.52, p=0.03) between anti-Ro52 and anti-Ro60 antibody levels (**Figure 1F**)—but not between anti-Ro52 or anti-Ro60 and anti-HRS titers (data not shown). When these parameters were reassessed in subsets of mice stratified by exposure to cigarette smoke versus air, there were trends toward increased production of anti-Ro52 and anti-Ro60 antibodies in serum and BALF of smoke-exposed mice that only achieved statistical significance for BALF anti-Ro52 antibody responses (**Supplementary Figure 2**).

To address the contribution of IFA and TLR7/8 activation and further distinguish HRS-induced immune responses against Ro52 and Ro60, we compared serum anti-Ro52 and anti-Ro60 antibody formation in mice immunized with HRS/IFA + R848 emulsions versus recombinant HRS alone. As shown in **Supplementary Figure 3**, serum anti-Ro60 antibody responses were somewhat higher in mice receiving HRS/IFA + R848 compared to recombinant HRS alone, even after normalization by anti-HRS antibody titers (p<0.01). In contrast, serum anti-Ro52 antibody titers were clearly lower in mice immunized with HRS/IFA + R848 relative to those immunized with recombinant HRS alone (p=0.02). Coupled with the variable ratios of anti-Ro52: anti-Ro60 antibody titers in individual mice, this divergent response to TLR7/8 activation again suggested differential regulation of HRS-induced anti-Ro52 versus anti-Ro60 immunity rather than a simple coordinated dose response to HRS immunization.

### Relative prevalence of anti-Ro52 and anti-Ro60 antibodies in patients with anti-Jo-1 antibodies

**Figure 2:**
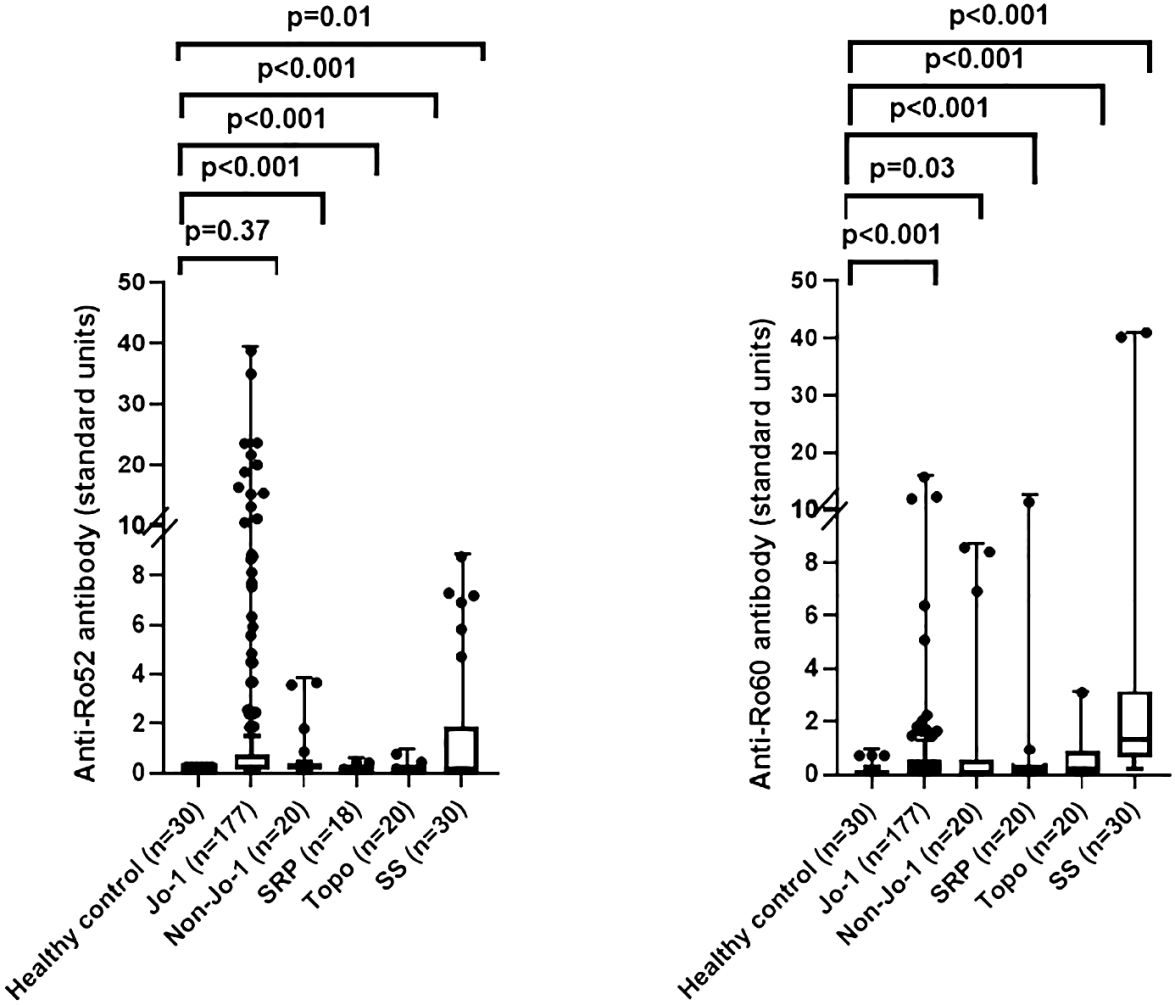
Comparison of anti-Ro52 and anti-Ro60 antibody levels in human sera. Box and whisker plots show relative anti-Ro52 and anti-Ro60 antibody titers (standardized units, y-axis) in patients with anti-Jo-1 antibodies versus healthy controls, primary Sjogren’s syndrome (SS), and patients with non-Jo-1 anti-tRNA synthetase, anti-signal recognition particle (SRP), or anti-topoisomerase I (anti-topo I) antibodies (x-axis). Individual patient values (in standardized units) are designated by dots, with median (IQR) antibody titers represented by horizontal bars and boxes. Listed p-values for statistically significant intergroup comparisons (designated by brackets) were determined by the Mann-Whitney U test.

Based on these findings in our murine model of HRS-induced myositis, we examined serum anti-Ro52 and anti-Ro60 antibody titers in 177 anti-Jo-1 antibody-positive patients as well as several control groups that included healthy volunteers (n=30) and patients with non-Jo-1 anti-tRNA synthetase (n=20), anti-SRP (n=20), or anti–topoisomerase I (n=20) antibodies. Using cutoff values based on the mean value plus 2 SD in healthy controls (0.3 for anti-Ro52 and 0.8 for anti-Ro60), the respective frequencies of anti-Ro52 and anti-Ro60 antibody positivity in different disease/autoantibody subsets were as follows: 36% (63/177) and 15% (27/177) of anti-Jo-1, 40% (8/20) and 20% (4/20) of non-Jo-1 anti-tRNA synthetase, 11% (2/18) and 10% (2/20) of anti-SRP, 10% (2/20), and 25% (5/20) of anti-topoisomerase I-positive patients—versus 30% (9/30) and 70% (21/30) of primary Sjogren’s syndrome patients. In contrast, none of the healthy control sera exceeded the defined thresholds for anti-Ro52 or anti-Ro60 antibody positivity (**Table 1**)—indicating that the increased frequency (relative to healthy controls) of anti-Ro52 and anti-Ro60 antibody positivity among Jo-1 positive patients was statistically significant (p<0.0001 and p=0.02 for anti-Ro52 and anti-Ro60 antibody positivity, respectively). Although median (IQR) values of anti-Ro52 and anti-Ro60 antibody titers (in standardized units) were both higher in anti-Jo-1-positive patients relative to healthy controls, these differences only achieved statistical significance for anti-Ro60 antibody titers (anti-Ro52: median 0.23 (0.13–0.75) vs median 0.21 (0.21–0.22) standard units, p=0.37; anti-Ro60: median 0.30 (0.00–0.58) vs median 0.00 (0.00–0.10) standard units, p<0.0001) (**Table 1**, **Figure 2**).

**Table 1:** Prevalence^1^ and relative titer of anti-Ro52 and Ro60 antibodies.

Antibody subset	Ro52 positive n (%)	Ro52 negative n (%)	Median (IQR)^2^	Ro60 positive n (%)	Ro60 negative n (%)	Median (IQR)^2^
Jo-1 (n=177)	63 (36)	114 (64)	0.23 (0.13-0.75)	27 (15)	150 (85)	0.30 (0.00-0.58)
Non-Jo-1 tRNA synthetase (n=20)	8 (40)	12 (60)	0.26 (0.24-0.42)	4 (20)	16 (80)	0.07 (0.00-0.55)
SRP (n=20)^3^	2 (11)	16 (89)	0.14 (0.14-0.15)	2 (10)	18 (90)	0.15 (0.07-0.40)
Topoisomerase 1 (n=20)	2 (10)	18 (90)	0.15 (0.14-0.15)	5 (25)	15 (75)	0.24 (0.02-0.89)
SS (n=30)	9 (30)	21 (70)	0.08 (0.07-1.88)	21 (70)	9 (30)	1.34 (0.63-3.19)
Healthy Control (n=30)	0 (0)	30 (100)	0.21 (0.21-0.22)	0 (0)	30 (100)	0.00 (0.00-0.10)

^1^Based on a standardized value of >0.3 units for Ro52 and 20.8 units for Ro60.

^2^Serum titers of anti-Ro52 and Ro60 antibodies, expressed in standardized units.

^3^Anti-Ro52 antibodies were measured in 18 of 20 patients who were positive for anti-SRP antibodies; anti-Ro60 antibody levels assessed in all 20 anti-SRP antibody-positive patients.

SD, standard deviation; ARS, aminoacyl tRNA synthetase; SRP, signal recognition particle; SS, Sjögren's syndrome.

**Figure 3:**
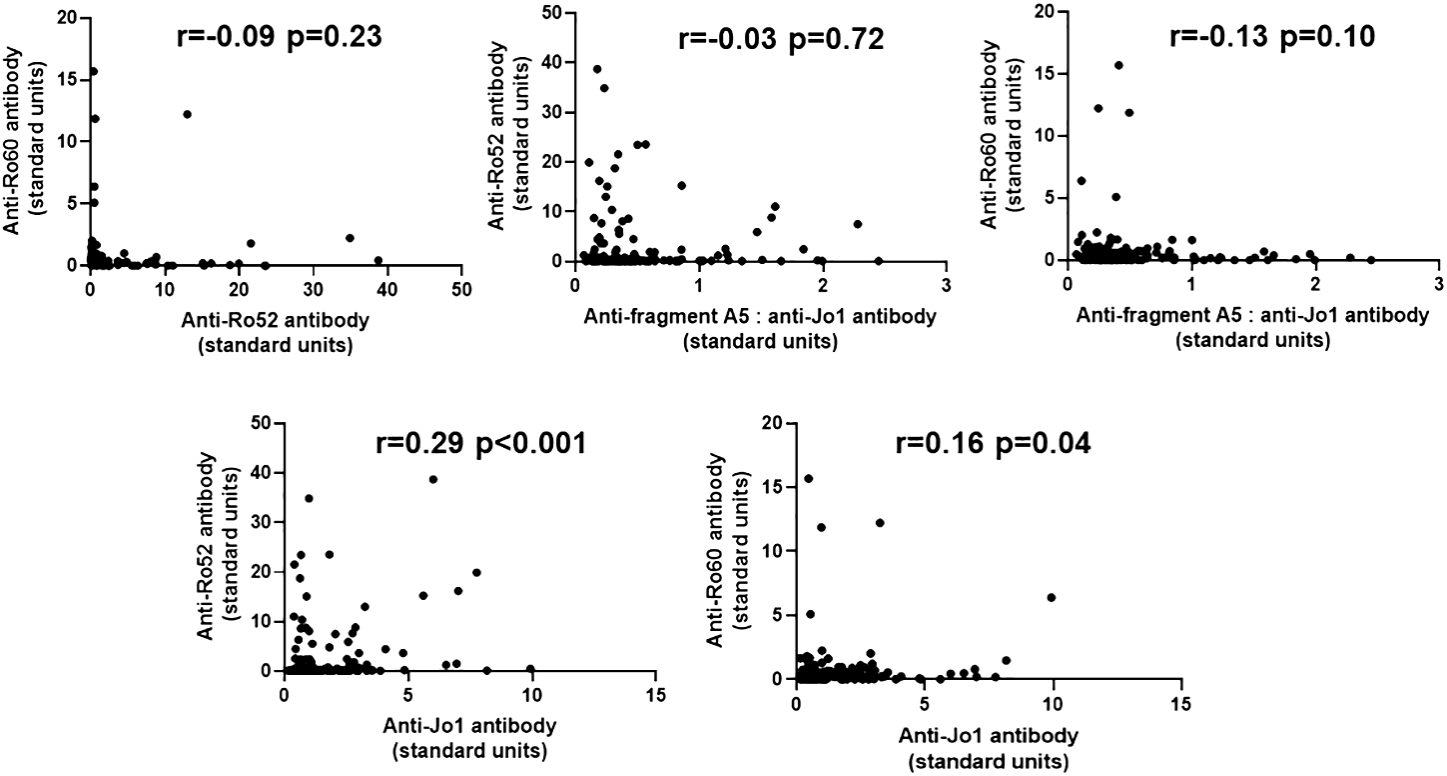
Relationship between anti-Ro52, anti-Ro60, and anti-Jo-1 antibody titers in Jo-1 antibody-positive patients. Scatter plots demonstrate the relationship between relative titers of anti-Ro52, anti-Ro60, and anti-Jo-1 antibodies (expressed as standard units derived from reference standard curves, as defined in Methods). Corresponding correlation coefficients (r-values) and p-values (determined by Spearman’s rank correlation analysis) are shown at the top of each panel.

To determine the potential quantitative relationship between levels of anti-Ro52, anti-Ro60, and anti-Jo-1 antibodies, we performed correlation analyses as shown in **Figure 3**. In short, there were no significant correlations between anti-Ro52, anti-Ro60, and anti-Jo-1 antibody titers, with the exception of anti-Ro52 and anti-Jo-1 antibodies that were weakly correlated (r=0.29, p<0.001). Moreover, competition ELISA experiments (**Supplementary Figure 4**) showed no cross reactivity between antibodies recognizing HRS versus Ro52 or Ro60, suggesting the possibility that qualitative differences in epitope recognition or site of the immune response might underlie the association between anti-Ro52, anti-Ro60, and anti-Jo-1 antibody formation.

### Clinical differences of anti-Jo1-positive patients based on anti-Ro52 and anti-Ro60 status

**Figure 4:**
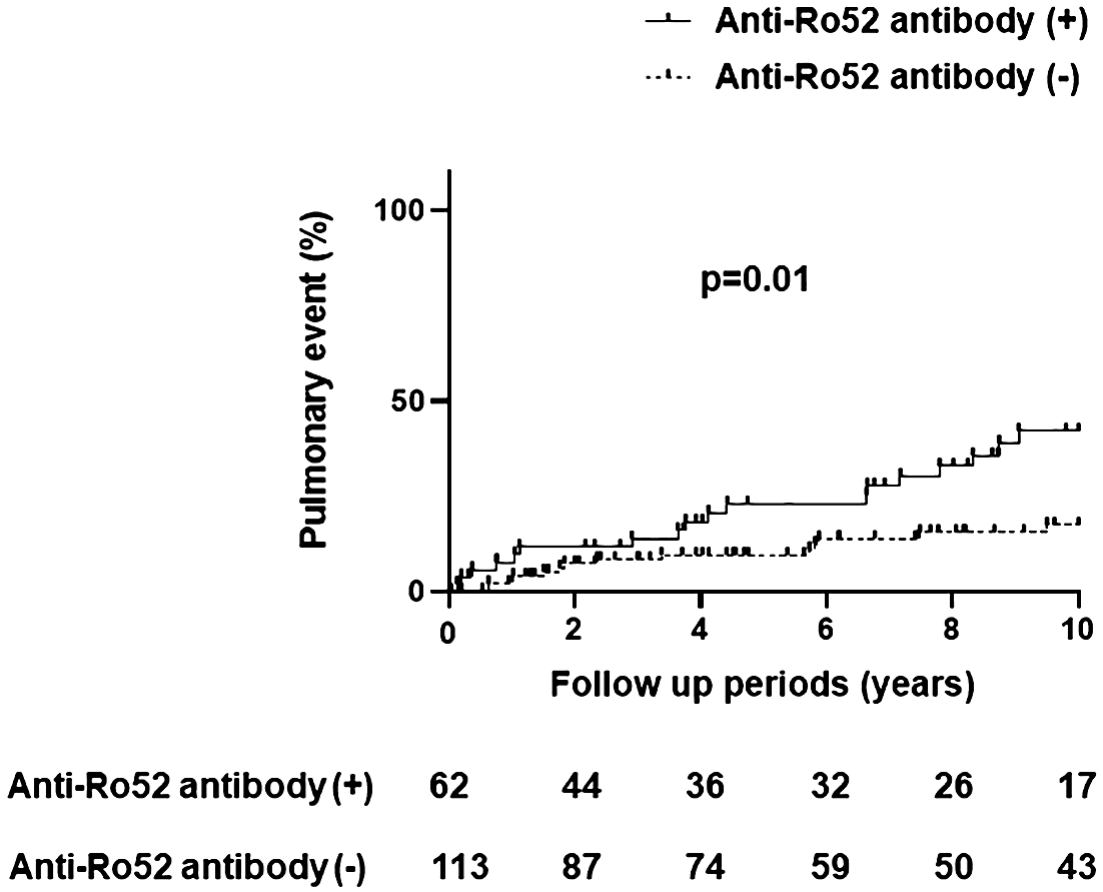
Pulmonary events in anti-Jo-1 antibody-positive patients with and without anti-Ro52 antibody positivity. The incidence of pulmonary events was assessed by Kaplan-Meier curves and log-rank tests, where the x-axis represents the follow-up period (10 years) and the y-axis represents pulmonary events (%) consisting of lung transplantation and/or death due to pulmonary causes.

**Table 2** demonstrates comparative clinical and laboratory findings between four groups of anti-Jo-1 antibody-positive patients stratified by the presence or absence of anti-Ro52 and anti-Ro60 antibodies. Age, race, sex, smoking history (ever vs. never), and disease subtype (myositis versus ILD without myositis versus overlap syndrome) did not significantly differ between these autoantibody subsets. However, other clinical features of anti-Jo-1 antibody-positive patients did vary based on anti-Ro52/anti-R60 antibody status. While Ro52(+) Ro60(-) patients had the highest frequency of ILD compared to the other groups, Ro52(-) Ro60(+) patients had the lowest frequency of ILD (p<0.01)—even compared to Ro52(-) Ro60(-) patients. Furthermore, Ro52(+) patients had more pulmonary events than Ro52(-) patients during the follow-up period, regardless of Ro60 status (p=0.01; **Figure 4**). Dry eye and dry mouth, on the other hand, were most frequent in Ro52(+) Ro60(+) patients and least frequent in Ro52(-) Ro60(-) patients (p=0.04 and 0.02, respectively, by Fisher’s exact test). Overall, anti-Ro60 antibody positivity was not associated with the presence of specific organ dysfunction, while patients with anti-Ro52 antibodies exhibited differences in the prevalence of ILD, sicca syndrome, and vascular dysfunction/Raynaud’s (**Supplementary Figure 5**).

**Table 2:** Clinical characteristics of anti-Ro52 and anti-Ro60 antibody-positive and negative patient in anti-Jo-1 antibody-positive cohort^1^.

	Ro52 (+) Ro60 n=50(%)	Ro52 (-) Ro60 n=14 (%)	Ro52 (+) Ro60 (+) n=13 (%)	Ro52 (-) Ro60 (-) n=100 (%)	p
Age (years)	52+12	51+19	4913	4813	0.27
Race (white/ Black)	45 4	9/4	13/0	88/12	0.07
Sex (male/female)	13/37	4/10	/ 10	36/64	0.55
Smoking (ever smoker)3	18 (45)	2 (22)	5 (42)	45 (52)	0.36
Myositis / ILD without myositis / Overlap syndrome4	35 /5/9	13 /0/1	10/1	87/3/9	0.22
Muscle weakness (n=135)5	38 (78)	11 (79)	11 (85)	75 (76)	0.91
Mechanic's hands (n=71)6	20 (44)	2 (17)	5 (39)	44 (46)	0.27
Classic DM rashes (n=55)	20 (42)	3 (21)	3 (23)	29 (29)	0.31
Arthritis/arthralgia (n=105)8	33 (70)	6 (43)	10 (77)	56 (57)	0.12
Vascular symptoms (n=77)9	26 (53)	4 (29)	9 (69)	38 (38)	0.06
Dysphagia (n=23)¹0	8 (17)	1 (7)	2 (15)	12 (12)	0.74
Dry eye (n=17)¹²	9 (20)	0 (0)	3 (25)	5 (5)	0.01
Dry mouth (n=20)¹3	9 (19)	0 (0)	4 (31)	7 (7)	0.01
ILD (n=142)¹	46 (96)	7 (54)	11 (85)	78 (80)	<0.01
Pulmonary event (n=32)¹4	14 (29)	0 (0)	4 (31)	14 (14)	0.03
Mortality (n=56)15	18 (37)	4 (29)	6 (46)	28 (28)	0.49
Follow-up periods (days)	2925 (1028-3650)	711 (280-3650)	2527 (18-3442)	2732 (1234-3650)	0.13
Anti-Jo-1 subfragment anti-FL Jo-1 ratios16					
Anti-fragment A2 : Anti-FL Jo-1 (IQR)	0.30 (0.20-0.69)	0.26 (0.15-0.68)	0.29 (0.17-0.45)	0.31 (0.23-0.43)	0.84
Anti-fragment A3 : Anti-FL Jo-1 (IQR)	0.36 (0.25-0.66)	0.26 (0.15-0.42)	0.33 (0.25-0.47)	0.32 (0.25-0.44)	0.43
Anti-fragment A4: Anti-FL Jo-1 (IQR)	0.38 (0.27-0.65)	0.25 (0.16-0.69)	0.39 (0.26-0.47)	0.32 (0.25-0.46)	0.37
Anti-fragment A5: Anti-FL Jo-1 (IQR)	0.35 (0.23-0.79)	0.24 (0.15-0.60)	0.34 (0.21-0.44)	0.32 (0.26-0.45)	0.31

^1^Age is presented as the mean (+S.D.) and laboratory markers as the median (IQR). P-values were established by Chi-square for categorical variables or the Kruskal-Wallis test.

^2^175 patients had evaluable baseline data for race.

^3^148 patients had evaluable baseline data for smoking history.

^4^175 patients had evaluable baseline data for disease classification.

^5^175 patients had evaluable baseline data for muscle weakness.

^6^165 patients had evaluable baseline data for mechanic's hands.

^7^174 patients had evaluable baseline data for classic DM rashes (heliotrope rash, Gottron papule/sign, V-neck sign, and/or shawl sign).

^8^173 patients had evaluable baseline data for arthritis / arthralgia.

^9^175 patients had evaluable baseline data for vascular symptoms.

^10^171 patients had evaluable baseline data for dysphagia.

^11^172 patients had evaluable baseline data for ILD.

^12^166 patients had evaluable baseline data for dry eye.

^13^171 patients had evaluable baseline data for dry mouth.

^14^175 patients had evaluable data for pulmonary event for follow-up periods.

^15^175 patients had evaluable data for mortality for follow-up periods.

^16^Jo-1 subfragment/MBP fusion proteins: A2-aa 1-60, A3=aa 1-90, A4=aa 1-120, A5=aa 1-151.

ILD, interstitial lung disease; DM, dermatomyositis; FL, full-length; IQR, interquartile range.

## Discussion

Through our established model of HRS-induced myositis that has allowed us to explore immunopathogenic mechanisms of the anti-synthetase syndrome, we have demonstrated that mice immunized with recombinant HRS develop antibodies against additional autoantigens including both Ro52 and Ro60. Despite their overlapping nomenclature, these distinct regulatory proteins occupy different subcellular niches and exert their biological effects through completely different mechanisms. While Ro52 is a nuclear and cytoplasmic TRIM family protein that is coordinately regulated by Type I IFN (33) and typically acts to limit inflammatory responses (33), Ro60 is an RNA-binding protein that is involved in processing misfolded RNA within the cytoplasm (33, 34). Immune responses targeting Ro52 have been linked to a number of autoimmune diseases including systemic lupus erythematosus (SLE), Sjogren’s syndrome, and idiopathic inflammatory myopathy (IIM) (33). Ro60, on the other hand, may be more selectively targeted, as isolated anti-Ro60 antibodies (without anti-Ro52 antibodies) are most commonly associated with SLE (35). Importantly, anti-Ro60 immune responses have only rarely been linked to IIM and/or the anti-synthetase syndrome.

Although many of the disease associations with anti-Ro52 (and to a lesser extent anti-Ro60) antibodies are well recognized, the immunological basis for concomitant targeting of these autoantigens and other “disease-specific” autoantigens remains undefined. As shown in our model of HRS-induced myositis, there is compelling evidence that autoantibodies targeting HRS, Ro52, and Ro60 are independently generated in BALF as well as serum—suggesting that the lung microenvironment may represent a primary site for breakdown of immune tolerance. Whether cigarette smoke exposure or alternative environmental insults provide additional stimulus for the breakdown of immune tolerance within the lung remains unclear, but our data showing trends toward increased anti-Ro52 and anti-Ro60 antibody formation in BALF/serum of smoke-exposed mice clearly warrants further study.

Of note, there is no clear correlation between levels of antibodies targeting HRS, Ro52, and Ro60 in the sera of HRS-immunized mice, an observation that is replicated in our human serum data and consistent with competition ELISA experiments in humans (**Supplementary Figure 4** and reference (36)) that do not reveal direct antibody cross-recognition of HRS (Jo-1) and Ro52/Ro60 proteins. Moreover, there is no obvious correlation between anti-Ro52/anti-Ro60 and anti-Jo-1 subfragment antibody responses in humans (**Table 2**), further disfavoring molecular mimicry as the basis for cross-recognition of these autoantigens. Also weighing against a potential molecular mimicry mechanism are previous studies showing no difference in Ro52 epitope recognition between Jo-1 antibody-positive and Jo-1 antibody-negative patients (36).

Based on the somewhat unexpected findings in our mouse model of HRS-induced myositis (most notably the development of anti-Ro60 antibodies), we sought to determine associations between the presence of anti-Ro52 and/or anti-Ro60 antibodies and the clinical phenotype of anti-Jo-1 (HRS) antibody-positive patients with the anti-synthetase or other overlap syndromes. In patients possessing both anti-Ro52 and anti-Jo-1 antibodies, we observed increased prevalence/severity of ILD, Raynaud’s phenomenon, and xerophthalmia as well as xerostomia. Our observations were consistent with previous studies demonstrating that serum levels of anti-Ro52 antibodies are linked to activity and/or severity of multiple disease manifestations in anti-Jo-1 antibody-positive individuals that include myositis, xerophthalmia/xerostomia, and ILD (37). In contrast, the presence of anti-Ro60 antibodies was not by itself associated with a distinct clinical phenotype in our large cohort of Jo-1 antibody-positive patients. Importantly, however, our data do suggest that immune responses targeting Ro60 may modulate the clinical phenotype of Jo-1 antibody-positive anti-synthetase syndrome, as Ro52(-) Ro60(+) patients have significantly less ILD than Ro52(-) Ro60(-) double antibody-negative patients. Similar trends are apparent for mechanic’s hands and arthritis/arthralgia (**Table 2**); in the case of classic DM rashes, the presence of anti-Ro60 antibodies is associated with diminished frequency of this extra-muscular complication, even within the anti-Ro52 antibody-positive subset (p>0.05).

Despite these collectively intriguing immunological and clinical observations regarding the relationship between humoral immune responses targeting Jo-1/HRS, Ro52, and/or Ro60 autoantigens, this work does have several limitations. First, laboratory-based studies in our unique model of HRS-induced myositis do not completely explain the mechanistic basis for these overlapping immune responses or fully define the role of Type I IFN signaling in promoting anti-Ro52 and/or anti-Ro60 immunity (given the relatively robust anti-Ro52 and anti-Ro60 immune responses to HRS immunization in the absence of IFA and the TLR7/8 agonist, R848; **Supplementary Figure 3**). Second, our analysis of serum samples derived from disease control groups does not reveal absolute specificity of anti-Ro52 or anti-Ro60 antibody responses for anti-Jo-1 antibody-positive individuals, raising additional questions regarding the immunological basis for overlapping antibody profiles. Third, our human studies are cross-sectional and subject to inherent limitations posed by retrospective studies (e.g., missing data)—a consideration that will require prospective, longitudinal data collection that may better capture the relationship between anti-Ro52/Ro60 immunity and evolving clinical features/disease outcomes in our large anti-Jo-1 antibody-positive cohort. Notwithstanding these limitations, the strengths of the current study—in which novel immunological observations from our highly translational murine model of HRS-induced myositis are merged with shifts in the clinical phenotype of humans with Jo-1 antibody-positive anti-synthetase syndrome–provide ample rationale for future studies exploring the clinical implications of anti-Ro52 and anti-Ro60 autoimmunity in IIM.

## Data availability statement

The original contributions presented in the study are included in the article/**Supplementary Material**. Further inquiries can be directed to the corresponding author.

## Ethics statement

The studies involving humans were approved by University of Pittsburgh Institutional Review Board. The studies were conducted in accordance with the local legislation and institutional requirements. The participants provided their written informed consent to participate in this study. The animal study was approved by University of Pittsburgh Institutional Animal Care and Use Committee. The study was conducted in accordance with the local legislation and institutional requirements.

## Author contributions

KY: Data curation, Formal analysis, Investigation, Writing – original draft, Writing – review & editing. QT: Data curation, Investigation, Writing – review & editing. PP: Data curation, Investigation, Writing – review & editing. DR: Investigation, Writing – review & editing. AG: Investigation, Writing – review & editing. RA: Writing – review & editing. CO: Data curation, Supervision, Writing – review & editing. DA: Conceptualization, Data curation, Formal analysis, Funding acquisition, Investigation, Resources, Supervision, Writing – original draft, Writing – review & editing.
