# Supplementary material for: Clinical features associated with the presence of anti-Ro52 and anti-Ro60 antibodies in Jo-1 antibody-positive anti-synthetase syndrome
Source: Front Immunol. 2024 Jun 4;15:1399451. doi: 10.3389/fimmu.2024.1399451 (PMC11183270; doi:10.3389/fimmu.2024.1399451)
Supplement: Supplementary file 1 [file DataSheet_1.pdf]

Online Supplemental Material. Supplementary Figure 1 illustrates the time course of HRS immunization experiments. While **Supplementary Figure 2** depicts autoantibody formation in HRS-immunized mice stratified by cigarette smoke exposure, **Supplementary Figure 3** compares the development of anti-Ro52 and anti-Ro60 antibodies in mice immunized with HRS/IFA + R848 (TLR7/8 agonist) versus HRS alone. **Supplementary Figure 4** represents competition ELISA experiments demonstrating that antibodies targeting Ro52, Ro60, and HRS are not cross-reactive. Finally, the box and whisker plots of **Supplementary Figure 5** show clinical associations of concomitant anti-Ro52 and anti-Jo-1 antibody-positivity, complementing data summarized in **Table 2**.

SUPPLEMENTARY FIGURE LEGENDS

Supplementary Figure 1. Experimental timeline for HRS-induced myositis. This schematic illustrates the period of cigarette smoke (versus air) exposure relative to administration of recombinant HRS/IFA emulsions and the TLR 7/8 agonist R848 in HRS-induced myositis.

Supplementary Figure 2. Anti-Ro52 and anti-Ro60 antibody formation in HRS-immunized mice stratified by cigarette smoke exposure. Panels A-B depict relative anti-Ro52 and anti-Ro60 antibody formation in serum (panel A) and BALF (panel B) of HRS- and PBS-immunized mice exposed to cigarette smoke (CS, 5 times/week) versus air alone over a two month period. Panel C demonstrates the ratio of BALF : serum anti-Ro52 and anti-Ro60 antibody formation in HRS-immunized mice exposed to cigarette smoke versus air. Relative antibody titers for individual mice (expressed as OD_{450} values, y-axes) are plotted against exposure category (x-axis); bars designate median values for n=10 cigarette-exposed mice and n=8 air-exposed mice. P-values (determined by Mann-Whitney U test) are listed above brackets designating specific intergroup comparisons.

Supplementary Figure 3. Contribution of TLR7/8 activation to anti-Ro52 and anti-Ro60 antibody formation. Panels A and B demonstrate anti-Ro52 (Panel A) and anti-Ro60 (Panel B) antibody responses in mice immunized intramuscularly with recombinant HRS/IFA + R848 (n=8) versus recombinant HRS alone (n=8). Dots correspond to adjusted OD_{450} values of anti-Ro52 or anti-Ro60 antibodies normalized by adjusted OD_{450} values of anti-HRS antibodies for individual mice; bars indicate median OD_{450} values for specified groups represented in the two plots. P-values determined by Mann-Whitney U test reflect inter-group comparisons encompassed by brackets.

Supplementary Figure 4. Competition ELISA and level of cross-reactivity between antibodies targeting HRS, Ro52, and Ro60. While panel A shows results of competition ELISAs involving pre-incubation of anti-Jo-1 antibody-positive human serum recognizing Ro52 with recombinant forms of HRS, panel B demonstrates the effects of competition between recombinant HRS and antibodies targeting Ro60. Serum dilutions and concentration range for competing antigens are listed below each panel. HA/MBP=human HRS aa 1-151 fused to MBP; Jo-1/MBP=full length human HRS fused to MBP; HRS=full-length human HRS (without MBP).

Supplementary Figure 5. Relationship between Anti-Ro52 antibody titers and clinical characteristics of anti-Jo-1 antibody-positive patients.

Box and whisker plots show the relationship between specific clinical findings (x-axis) and anti-Ro52 antibody titer (y-axis). While bars indicate median antibody titers (standardized units), boxes and whiskers reflect IQRs and maximum/minimum values, respectively; p-values based on Mann Whitney U testing are listed above each comparison.
