# Supplementary figures and images for: Clinical features associated with the presence of anti-Ro52 and anti-Ro60 antibodies in Jo-1 antibody-positive anti-synthetase syndrome
Source: Front Immunol. 2024 Jun 4;15:1399451. doi: 10.3389/fimmu.2024.1399451 (PMC11183270; doi:10.3389/fimmu.2024.1399451)

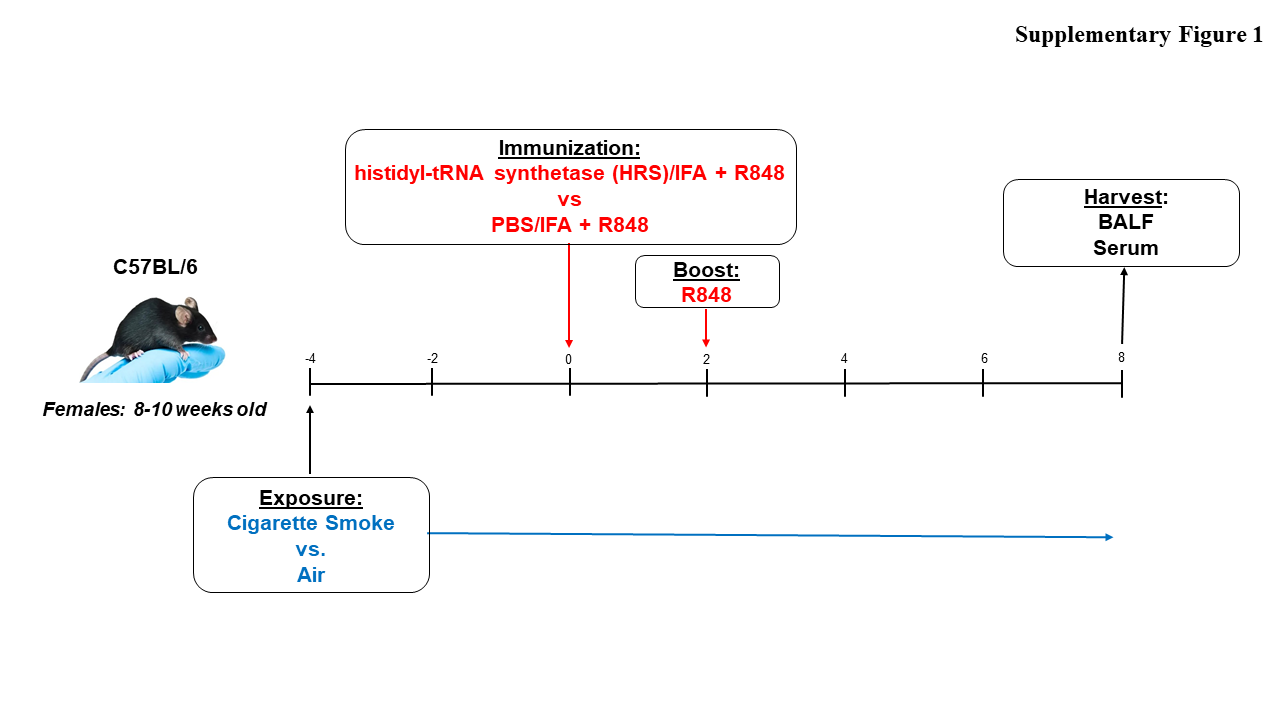

Supplement: Supplementary file 2 [file Image_1.tif]

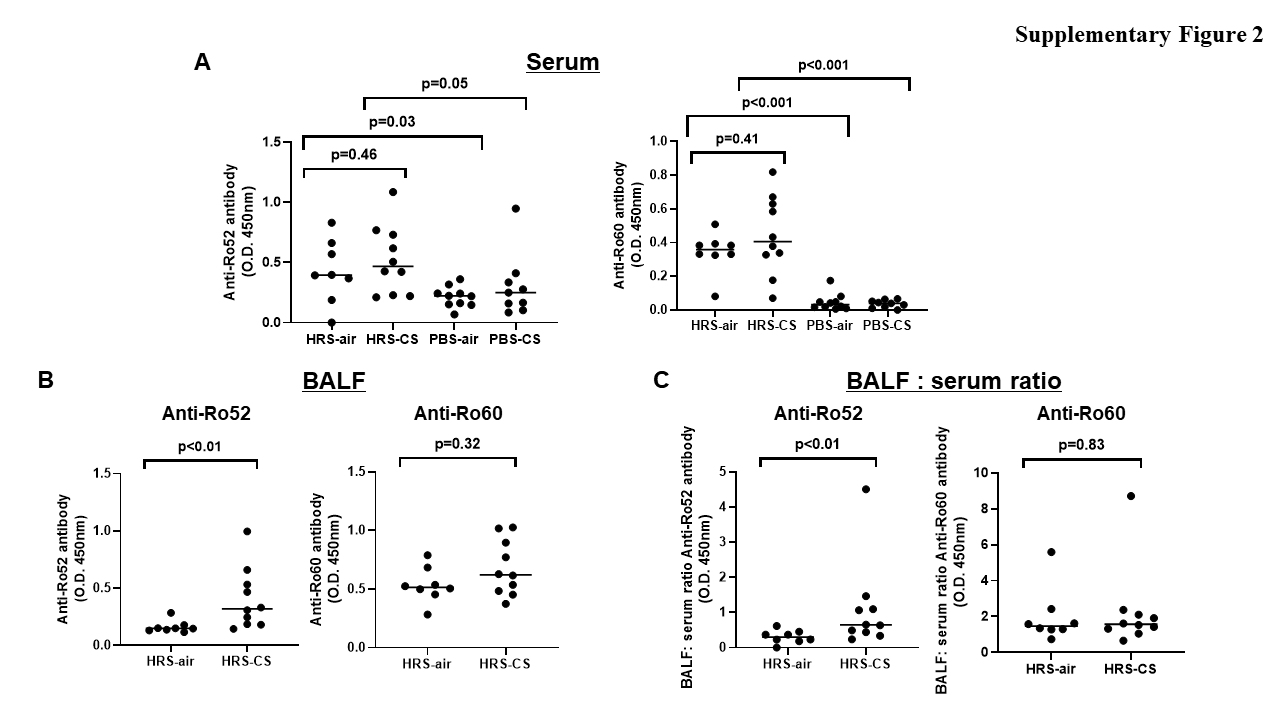

Supplement: Supplementary file 3 [file Image_2.tif]

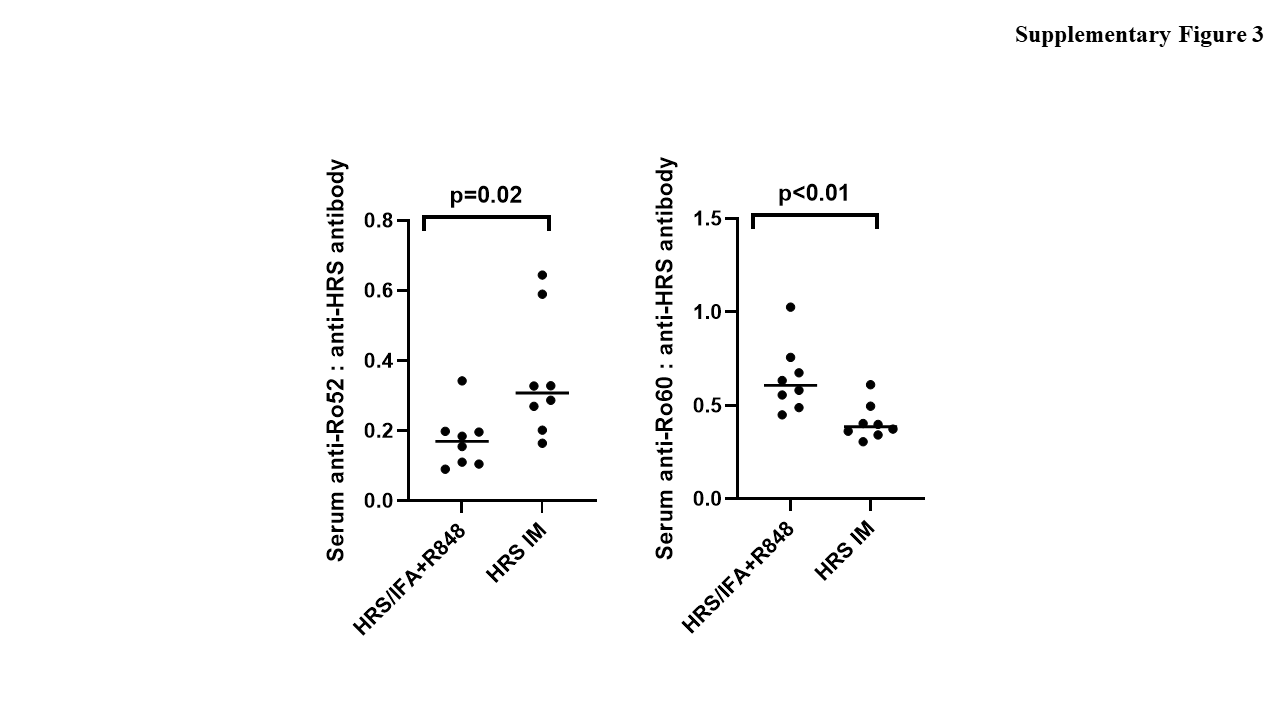

Supplement: Supplementary file 4 [file Image_3.tif]

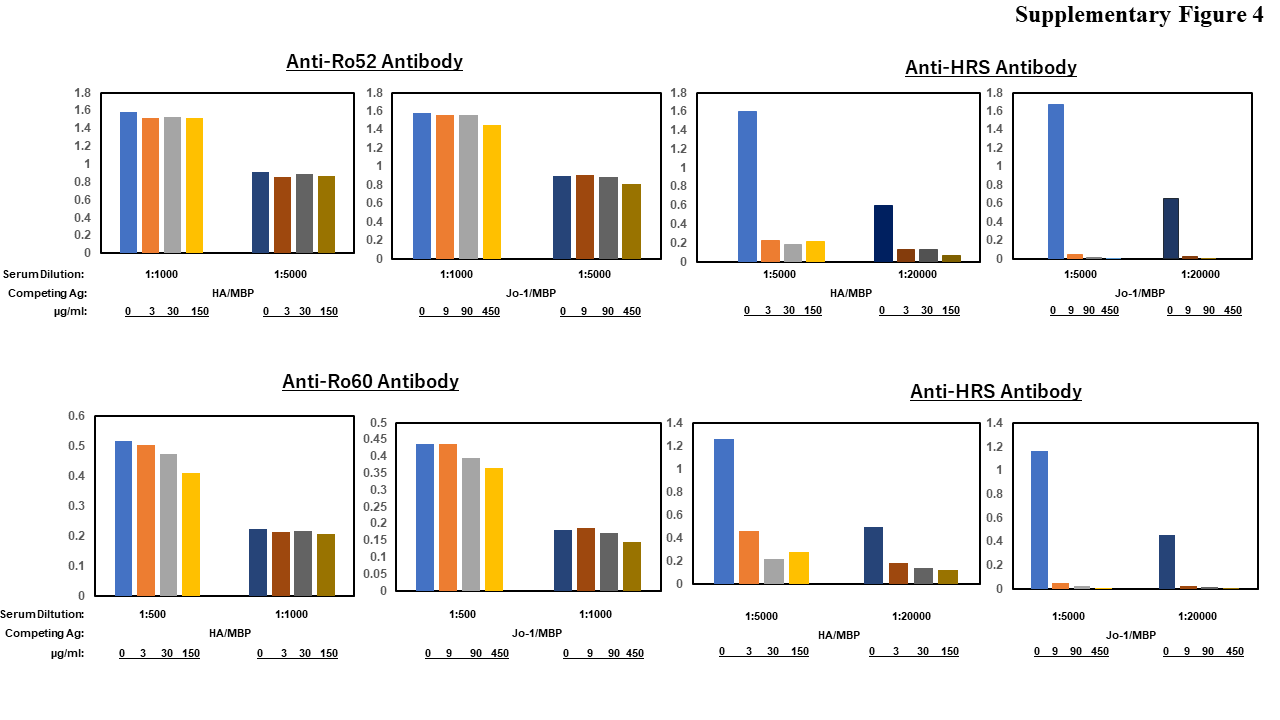

Supplement: Supplementary file 5 [file Image_4.tif]

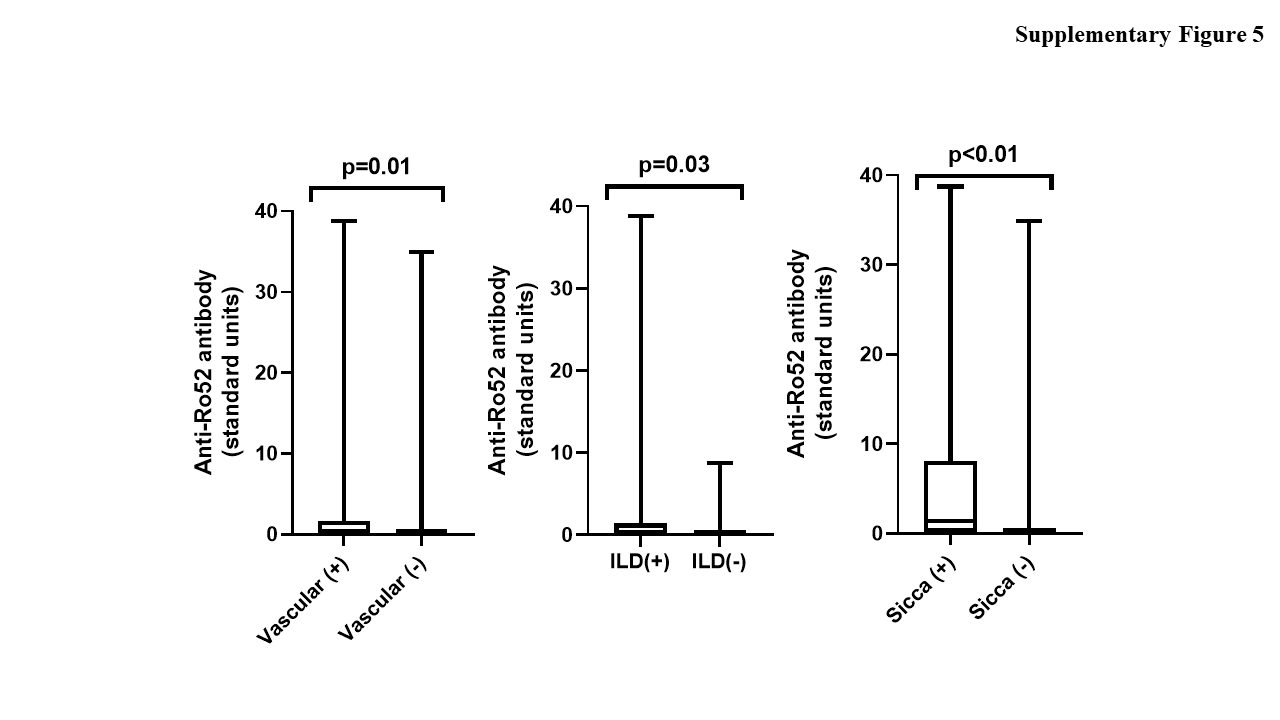

Supplement: Supplementary file 6 [file Image_5.tif]
